# Supplementary material for: Rapid floral syndrome convergence in Penstemon through independent genetic variation
Source: bioRxiv. 2026 Jul 24:2026.07.21.739878. Preprint. [Version 1] doi: 10.64898/2026.07.21.739878 (PMC13419788; doi:10.64898/2026.07.21.739878)
Supplement: Supplement 1 [file media-1.pdf]

Supplemental Materials and Methods

Phenotyping

We measured the following floral dimension traits on whole flowers using digital calipers: floral tube length (length from the base of the flower to the tube opening), tube width (width at the tube opening), long stamen length (length from the base of the flower to the end of the lateral stamens), short stamen length (length from the base of the flower to the end of the ventral stamens), and style length (length from the base of the flower to the end of the pistil). We measured nectar volume in microliters from flowers on the first day of opening using 5 µl micro-capillary tubes (Drummond Scientific, Broomali, PA, USA). We imaged the nectaries found at the base of the short stamen filaments alongside a millimeter ruler under a Dino-Lite Premier digital microscope (Dino-Lite, Torrance, CA, USA) set to 20x magnification and measured nectary area from the resulting photographs using *FIJI* (Schindelin et al., 2012).

We extracted anthocyanidin pigments from pressed floral tissue using an isoamyl alcohol extraction protocol (Harborne, 1984) and separated pigments using thin layer chromatography on Cellulose F glass plates (Millipore, Burlington, MA, USA), following Stevens et al. (2023). We compared extracted pigments to the standard anthocyanidins pelargonidin, cyanidin, and delphinidin and scored for presence of each type. Note that methylated anthocyanidin pigments are not produced by *Penstemon* species (Wessinger & Rausher, 2015). We assigned each accession a numeric value based on their qualitative anthocyanidin content that reflects the number of hydroxyl groups on the central B-ring of the anthocyanidin pigment: 1=pelargonidin, 2=cyanidin, 3=delphinidin. Individuals producing a combination of pigments were assigned the average of the two values (e.g., 2.5 assigned to individuals producing cyanidin and delphinidin).

We used reflectance spectroscopy to measure the flower color of the upper petal lobe surface with a UV-vis spectrometer (FLAME-S-UV-VIS, Ocean Insight, Dunedin, FL, USA), equipped with a Y-shaped reflection probe (R400-7-UV-VIS, Ocean Insight) that supplied light from a deuterium/tungsten light source (DH-200-BAL, Ocean Insight). We calibrated reflectance measurements with a Spectralon diffuse reflectance standard (WS-1, Ocean Insight) before recording reflectance data from 300 to 800 nm using OceanView software (Ocean Insight) in a darkened room with no overhead lighting. We sandwiched petal tissue between two clean glass microscope slides to eliminate spectral variation due to petal curvature and then measured reflectance with the reflectance probe at a 45-degree angle relative to the sample. We performed these measurements on 25 of the 34 sampled individuals.

We processed raw reflectance spectra using the R package *pavo* (Maia et al., 2019). We calculated hue and chroma using segment classification (Smith, 2014). Hue is calculated in degrees – a circular metric – and because the values in our dataset bridge the 0°/360° boundary, we calculated an "adjusted hue" value by by adding 90° to each hue value. We also estimated the detectability of each floral reflectance spectrum to bees vs. hummingbirds by comparing chromatic contrast values under a honeybee visual model vs. an average avian violet sensitive model (to represent hummingbird vision) using *pavo.* We calculated quantum catch for each photoreceptor against a background spectrum measured from a *Penstemon* leaf using the *pavo* function *vismodel*. We then used the *pavo* function *coldist* to calculate distances between colors in each pollinators' visual space under a receptor-noise model (Vorobyev & Osorio, 1998). This model yields chromatic contrast values in units of Just Noticeable Differences (JNDs), where values below 1 are assumed not to be visually detectable and increasing JND values indicate the color is increasingly detectable.

Whole genome resequencing

We extracted DNA from fresh or silica-dried leaf tissue using a modified CTAB protocol. Whole genome Illumina library preparations were generated using the Illumina DNA Prep kit and resulting libraries were sequenced to ~5x depth using 150-bp paired-end reads generated on the NovaSeq X platform (Illumina, San Diego, CA, USA) at the Duke University Sequencing and Genomic Technologies research core facility.

Using *fastp* version 0.23.2 (Chen et al., 2018), we trimmed adapters and poly-x on the 3'-ends of reads, corrected bases for overlapping reads, and filtered out unpaired reads or those less than 30 bp. We then checked filtered sequence quality using *fastqc* version 0.11.8 (Andrews et al., 2010). We mapped our filtered reads to the annotated *P. eatonii* reference genome (Jarvis et al., 2025) using *bwa mem* version 0.7.17 (Li, 2013), marked and removed duplicated reads using *samtools* version 1.51.1 (Li et al., 2009), and clipped overlapping paired-end reads using *bamutil* clipOverlap (Jun et al., 2015).

We generated an all sites vcf file using *bcftools* (Li, 2011) in single-sample calling mode, using a minimum mapping quality of 30, a minimum base quality of 20, and excluding sites within 5 bp of an indel. We excluded sites within annotated repeats using *bcftools* and retained only those sites that mapped to the 8 major chromosomes of the reference genome. As a final filtering step, we filtered on minimum genotype call quality (2), maximum number of alleles (2), minimum read depth (2), maximum mean depth (16), maximum per-site proportion of heterozygote calls (75%), and maximum per-site proportion of missing individuals (10%) using *vcftools* version 0.1.17 (Danecek et al., 2011).

Tree inference

We generated whole-genome consensus sequences using the *consensus* function of *bcftools*. We then generated two sets of sequence alignments: 10 kb non-overlapping windows and coding sequences (CDS) using custom python scripts reported in Stone and Wessinger (2024). We then inferred trees for each sequence alignment using *IQ-TREE* version 2.1.2 (Nguyen et al., 2015) under a general time reversal model, allowing invariable sites and rate variation across sites (-m GTR+I+G). We used *ASTRAL-III* version 5.7.1 (Zhang et al., 2018) to infer a CDS-based species tree from the collection of CDS trees and a 10 kb window-based species tree from the collection of 10 kb window trees. We rooted each tree using the outgroup taxon *P. palmeri*. We calculated gene concordance factors on each species tree using *IQ-TREE* (Minh et al., 2020).

We inferred relative divergence times for the 10 kb window-based ASTRAL tree using a penalized likelihood approach implemented in *treePL* version 1.0 (Smith & O’Meara, 2012). Specifically, we first used *treePL* to test optimal settings using the 'prime' mode and then ran a final analysis using the 'thorough' mode and the following program settings: opt=5, optad=5, and optcvad=2. We used this ultrametric tree for all phylogenetic comparative analyses.

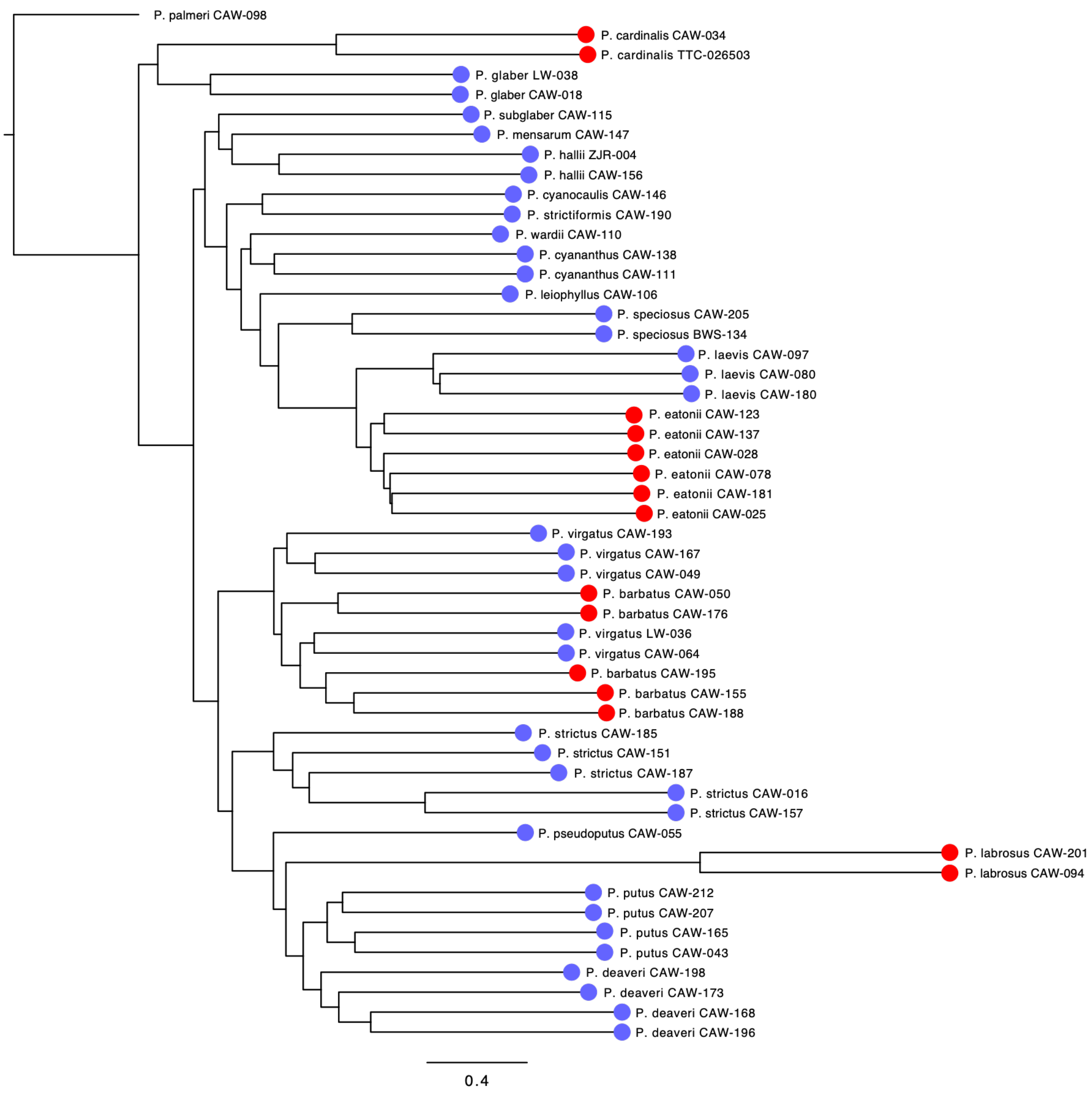

Figure S1. Phylogenetic relationships between accessions inferred using a species tree approach based on CDS regions. Purple: bee syndrome species; red: hummingbird syndrome species.

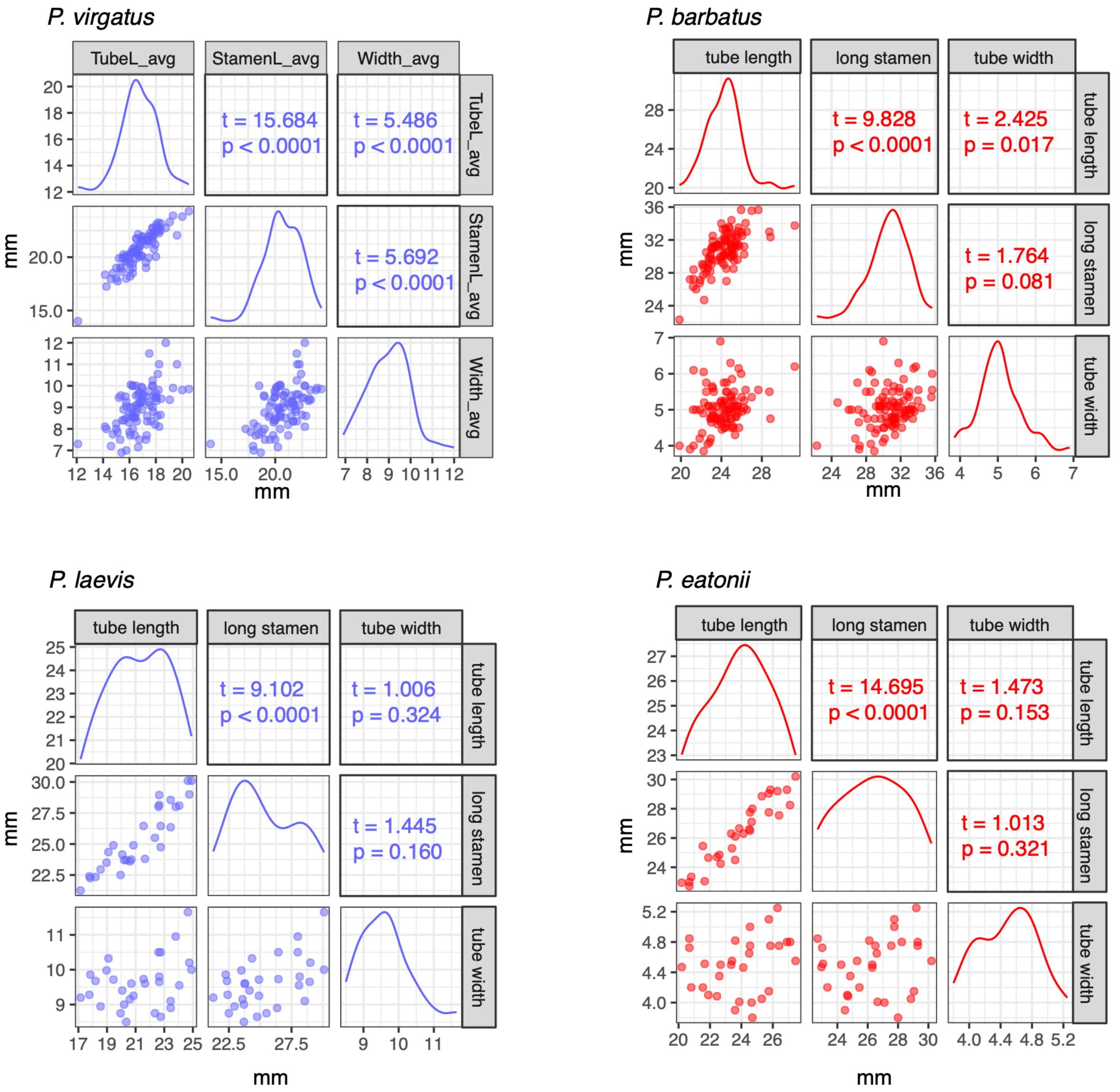

Figure S2. Relationships among phenotypes measured with populations of two bee syndrome species (*P. virgatus* and *P. laevis*) and two hummingbird syndrome species (*P. barbatus* and *P. eatonii*). Reported are t- and p-values from linear models described in the main text.

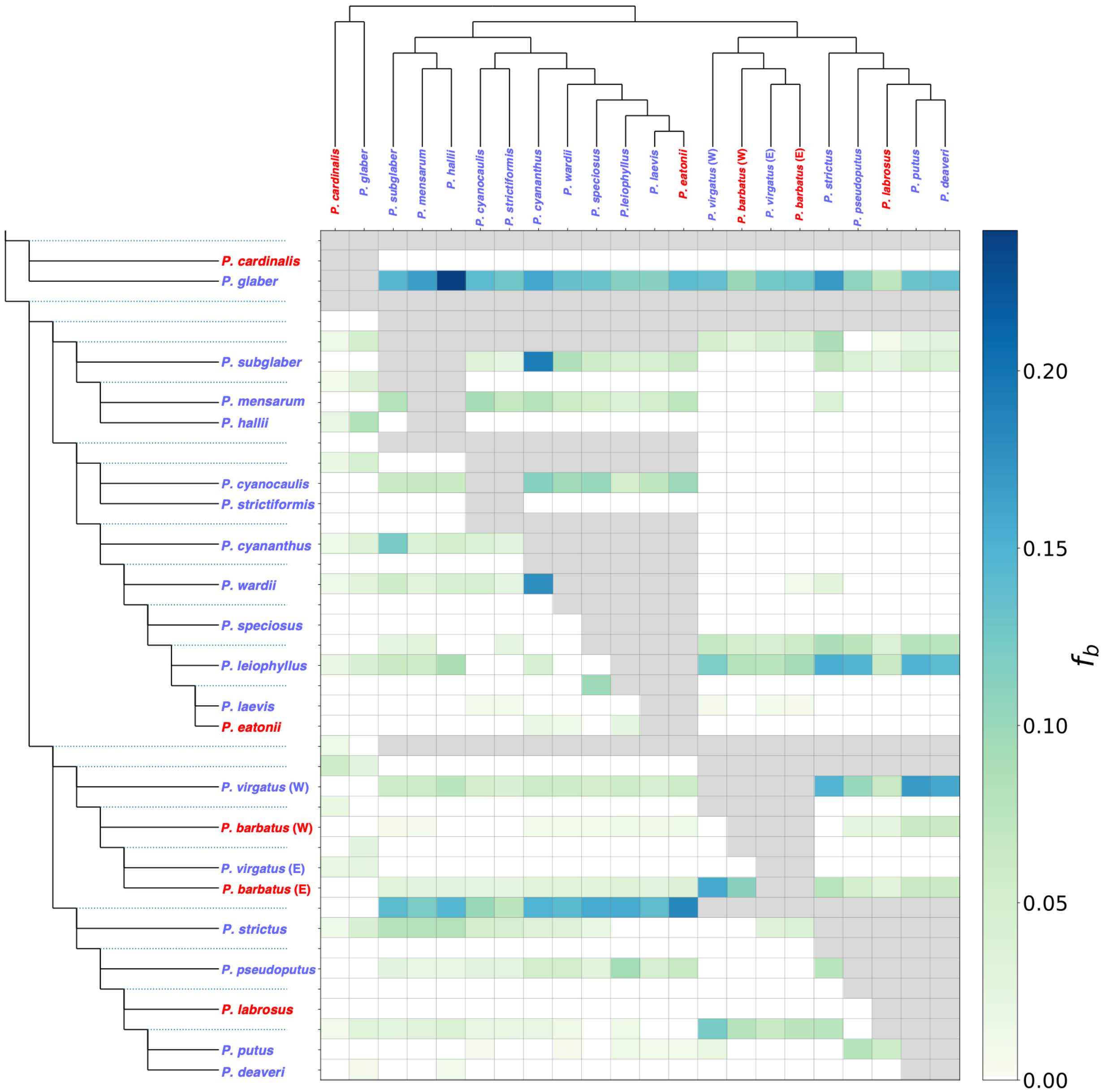

Figure S3. Results of *f*-branch analysis testing for excess allele sharing between branches and tips in the species tree, which is interpreted as evidence for introgression between species and/or clades. The species tree used here is the ASTRAL tree based on 10 kb genomic windows. Red: hummingbird syndrome species, purple: bee syndrome species.

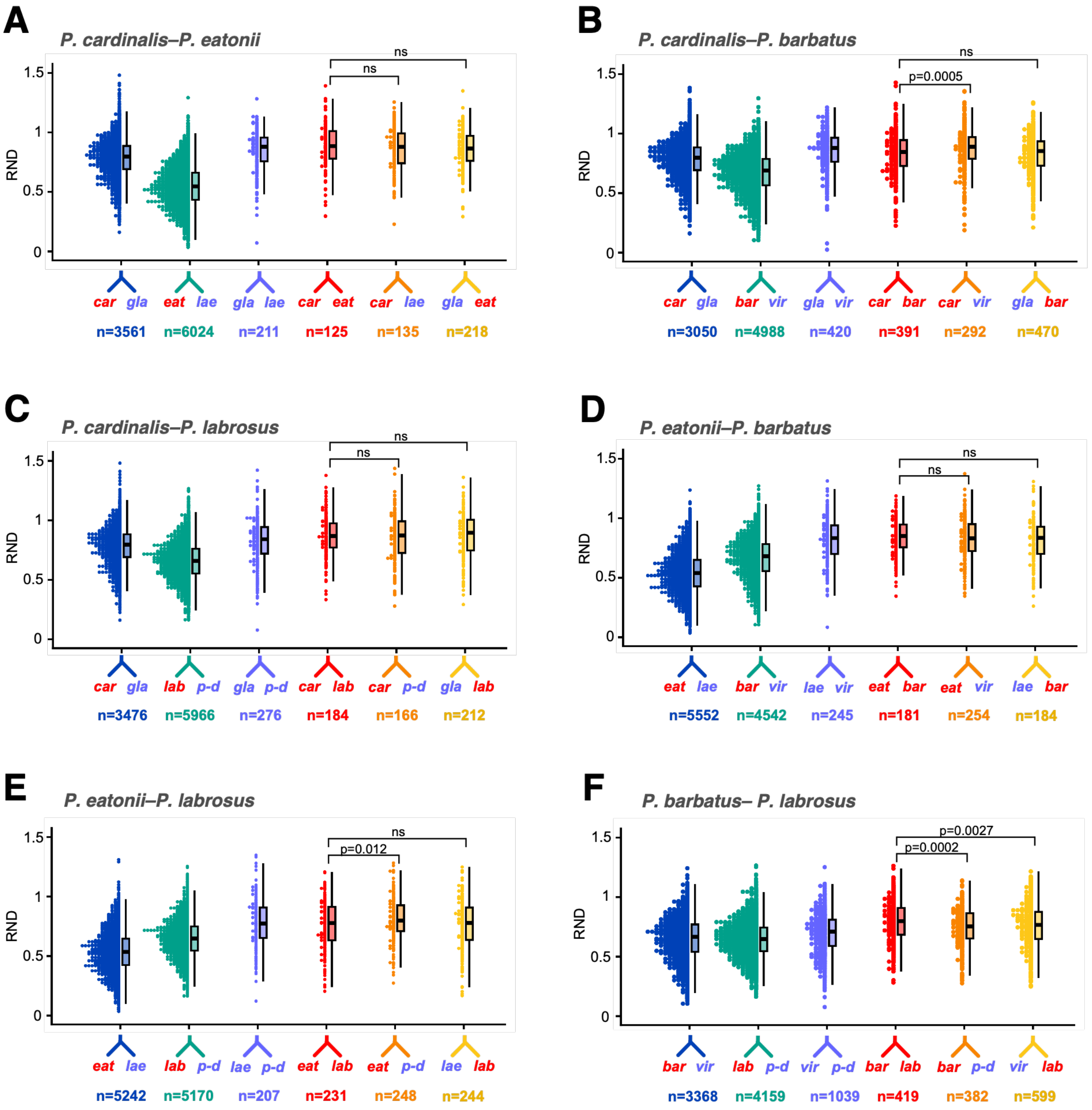

Figure S4. Distributions of relative node depth (RND) for 10 kb regions of the genome showing allele sharing between the following taxa: H1-B1 (navy), H2-B2 (green), B1-B2 (purple), H1-H2 (red), H1-B2 (orange), and H2-B1 (yellow). Significance of Mann-Whitney U tests are reported. Ns: $p > 0.05$.

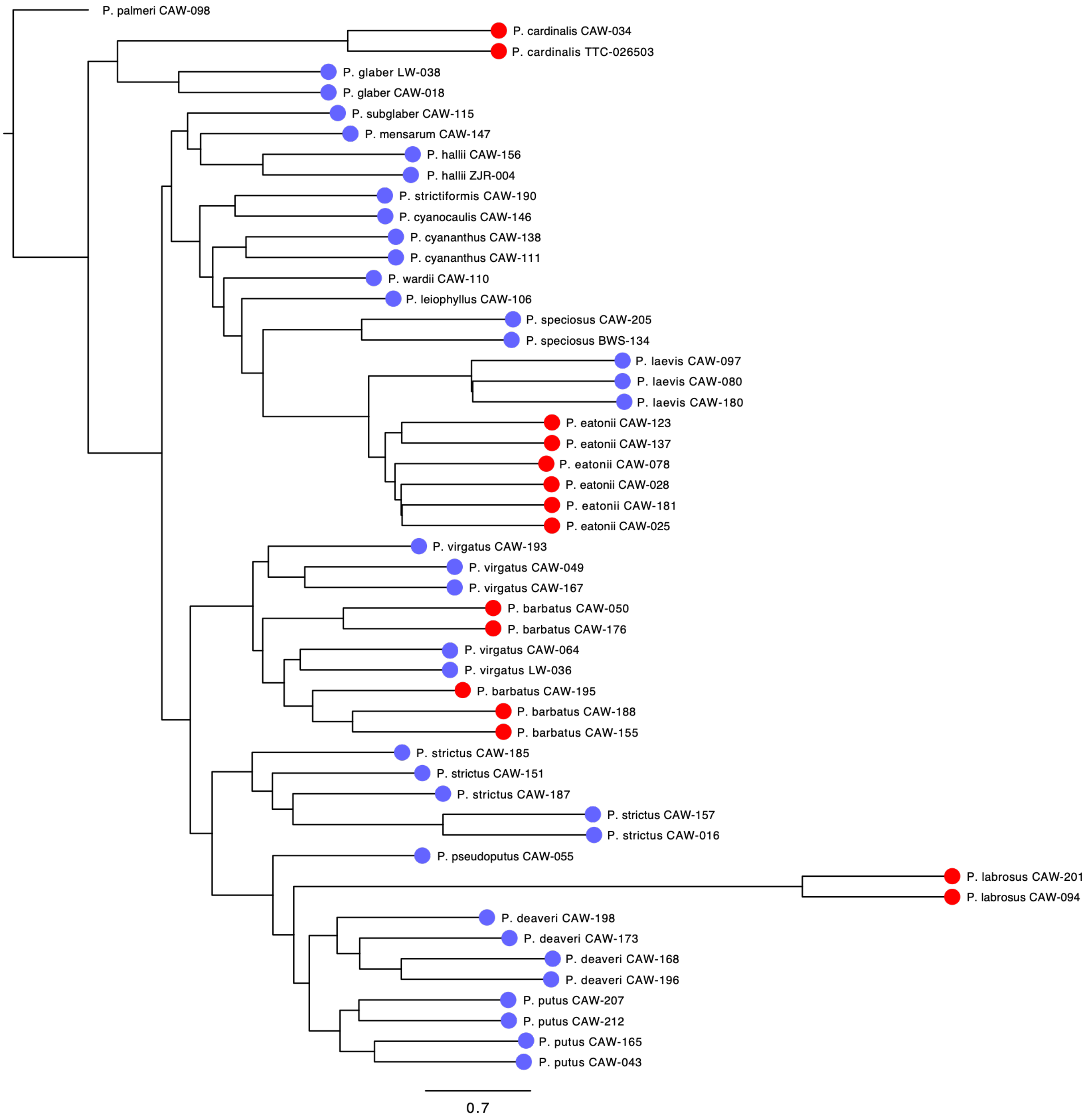

Figure S5. Phylogenetic relationships between accessions inferred using a species tree approach based on 10 kb window regions and using the *P. barbatus* reference genome. Purple: bee syndrome species; red: hummingbird syndrome species.

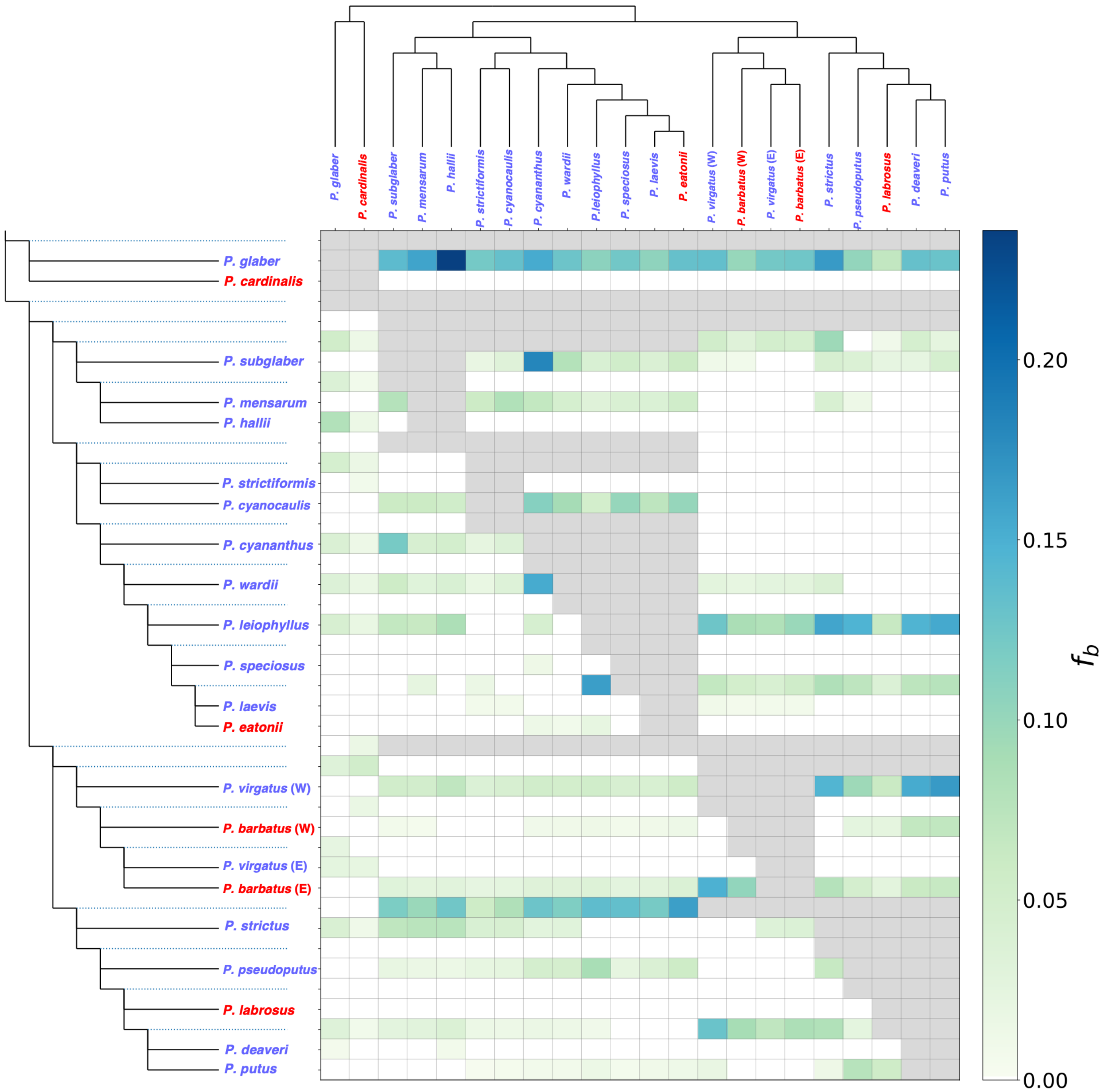

Figure S6. Results of *f*-branch analysis testing for excess allele sharing between branches and tips in the species tree using the *P. barbatus* reference genome.The species tree used here is the ASTRAL tree based on 10 kb genomic windows. Red: hummingbird syndrome species, purple: bee syndrome species.

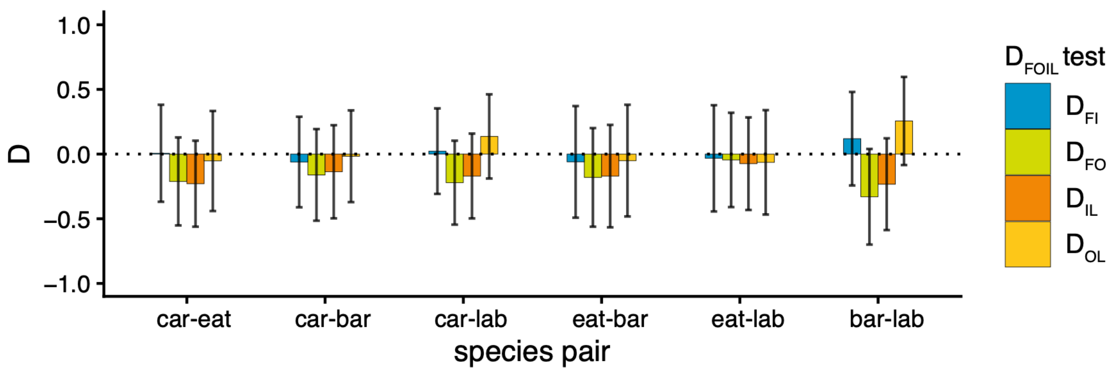

Figure S7. Results of D_{FOIL} tests for all six pairwise tests of introgression, using the *P. barbatus* reference genome.

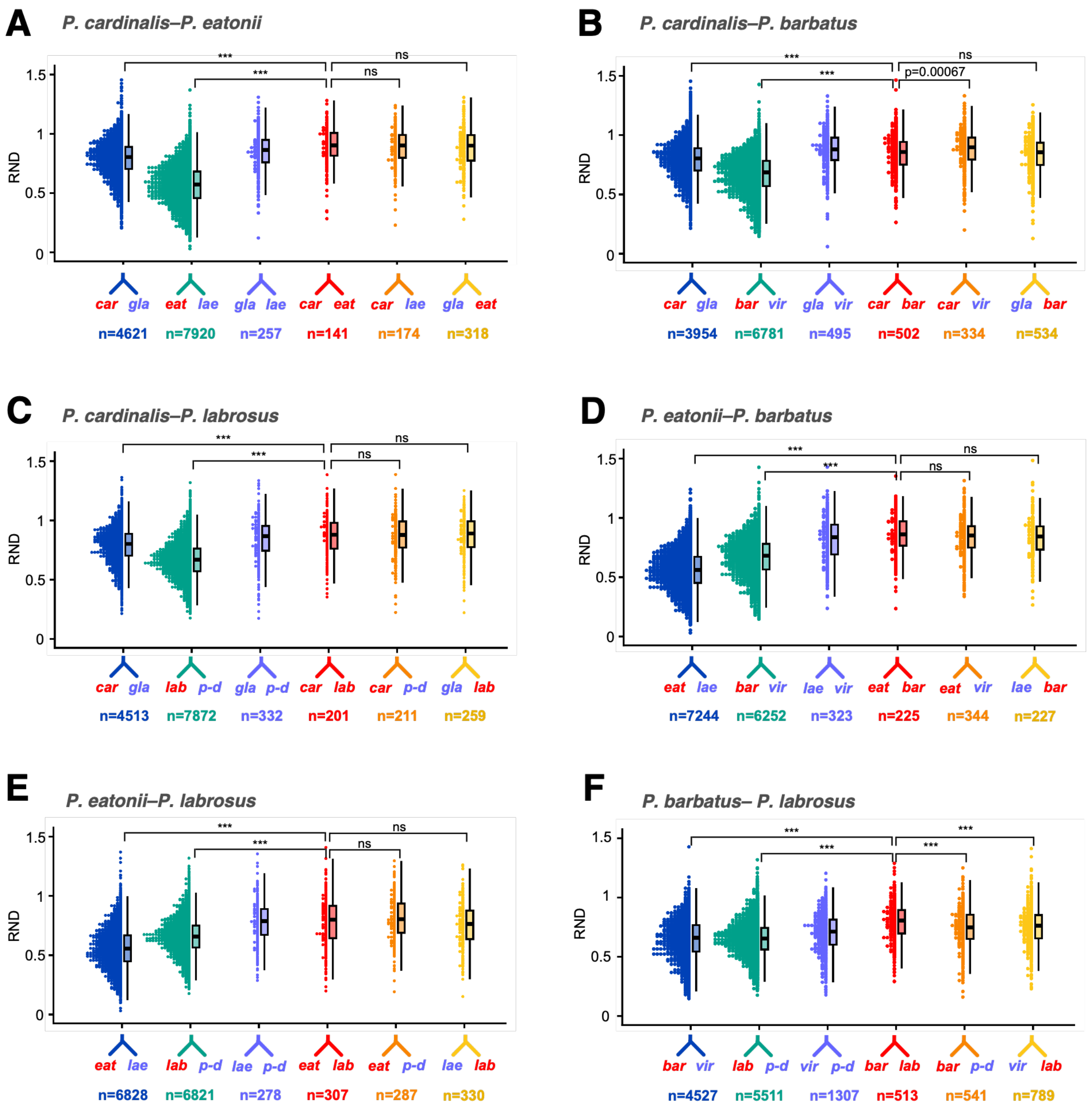

Figure S8. Distributions of relative node depth (RND) for 10 kb regions of the genome (*P. barbatus* reference genome) showing allele sharing between the following taxa: H1-B1 (navy), H2-B2 (green), B1-B2 (purple), H1-H2 (red), H1-B2 (orange), and H2-B1 (yellow). Significance of Mann-Whitney U tests are reported. Ns: $p > 0.05$, ***: $p < 0.0001$.

Table S1. Collection information for *Penstemon* samples used in this study. BWS: Benjamin Stone, CAW: Carolyn Wessinger; COLO: University of Colorado Museum of Natural History; KANU: R.L. McGregor Herbarium at University of Kansas; LCW: Lucas Wheeler; MJ: Matthew Johnson; TTC: E.L. Reed Herbarium at Texas Tech University; ZJR: Zachary Radford. Asterisks indicate accessions used in 5-taxon tests for introgression.

**Population identifier**	**Species determination (abbreviation in Figure 1)**	**Locality of population**	**Collector**	**Herbarium deposited**	**Phenotyped**
BWS-134	*P. speciosus*	Mono Pass, CA	BWS	KANU	no
CAW-016	P. strictus (str-016)	Victor, CO	CAW	KANU	yes
CAW-018*	*P. glaber*	Midland, CO	CAW	KANU	no
CAW-025	*P. eatonii*	Cortez, CO	CAW	KANU	no
CAW-028	*P. eatonii* (eat-028)	McDowell, AZ	CAW	KANU	yes
CAW-034*	*P. cardinalis* (car-034)	Bonito Lake, NM	CAW	KANU	yes
CAW-043*	*P. putus*	Pine, AZ	CAW	KANU	no
CAW-049*	*P. virgatus* (vir-049)	Kendrick Park, AZ	CAW	KANU	yes
CAW-050	*P. barbatus* (bar-050)	Flagstaff, AZ	CAW	KANU	yes
CAW-055	*P. pseudoputus* (psp-055)	Kaibab Plateau, AZ	CAW	KANU	yes
CAW-064	*P. virgatus* (vir-064)	Cloudcroft, NM	CAW	KANU	yes
CAW-078	*P. eatonii*	Mt. Carmel Jct., UT	CAW	KANU	no
CAW-080*	*P. laevis*	Mt. Carmel Jct., UT	CAW	KANU	no
CAW-094*	*P. labrosus* (lab-094)	Big Bear Lake, CA	CAW	KANU	yes
CAW-097	*P. laevis* (lae-097)	Rosy Canyon, AZ	CAW	KANU	yes
CAW-098*	*P. palmeri*	Rosy Canyon, AZ	CAW	KANU	no
CAW-106	*P. leiophyllus* (lei-106)	Cedar Canyon, UT	CAW	KANU	yes
CAW-110	*P. wardii*	Soldier Canyon, UT	CAW	KANU	no
CAW-111	*P. cyananthus*	Nebo Loop, UT	CAW	KANU	no
CAW-115	*P. subglaber* (sub-115)	Tucker, UT	CAW	KANU	yes
CAW-123*	*P. eatonii*	Antimony, UT	CAW	KANU	no
CAW-137	*P. eatonii*	Three Creeks, UT	CAW	KANU	no
CAW-138	*P. cyananthus*	Three Creeks, UT	CAW	KANU	no
CAW-146	*P. cyanocaulis*	Devil's Kitchen, CO	CAW	KANU	no
CAW-147	*P. mensarum* (men-147)	Powderhorn Ski, CO	CAW	KANU	yes
CAW-151	*P. comarrhenus*	Mesa Creek, CO	CAW	KANU	no
CAW-155*	*P. barbatus*	Cottonwood Pass, CO	CAW	KANU	no
CAW-156	*P. hallii*	Fremont Pass, CO	CAW	KANU	no
CAW-157	*P. strictus* (str-157)	Keystone, CO	CAW	KANU	yes
CAW-165	*P. putus* (put-165)	Forest Lakes, AZ	CAW	KANU	yes
CAW-167	*P. virgatus* (vir-167)	Overgaard, AZ	CAW	KANU	yes
CAW-168	*P. deaveri*	Sheeps Crossing, AZ	CAW	KANU	no
CAW-173	*P. deaveri* (dea-173)	Willow Creek, NM	CAW	KANU	yes
CAW-176	*P. barbatus* (bar-176)	Pinos Altos, NM	CAW	KANU	yes
CAW-180	*P. laevis*	Rosy Canyon, UT	CAW	KANU	no
CAW-181	*P. eatonii* (eat-181)	Gooseberry Mesa, UT	CAW	KANU	yes
CAW-185	*P. strictus*	Eagles Nest Lake, NM	CAW	KANU	no
CAW-187	*P. strictus* (str-187)	Horca, CO	CAW	KANU	yes
CAW-188	*P. barbatus* (bar-188)	La Manga Pass, CO	CAW	KANU	yes
CAW-190	*P. strictiformis* (stf-190)	Llaves, NM	CAW	KANU	yes
CAW-193	*P. virgatus* (vir-193)	Mt. Taylor, NM	CAW	KANU	yes
CAW-195	*P. barbatus* (bar-195)	Zuni Canyon, NM	CAW	KANU	yes
CAW-196	*P. deaveri* (dea-196)	Gallo Peak, NM	CAW	KANU	yes
CAW-198	*P. deaveri* (dea-198)	Taylor Peak, NM	CAW	KANU	yes
CAW-201	*P. labrosus* (lab-201)	Big Bear Lake, CA	CAW	KANU	yes
CAW-205	*P. speciosus* (spe-205)	Sunriver, OR	CAW	KANU	yes
CAW-207	*P. putus* (vir-207)	Humphreys Peak, AZ	CAW	KANU	yes
CAW-212	*P. putus*	Sycamore Falls, AZ	CAW	KANU	no
LW-036	*P. virgatus*	Four Mile Canyon, CO	LW	COLO	no
LW-038	*P. glaber* (gla-038)	Brainard Lake, CO	LW	COLO	yes
TTC026503	*P. cardinalis*	Guadalupe Mtns, TX	MJ	TTC	no
ZJR-004	*P. hallii* (hal-004)	Guanella Pass, CO	ZJR	KANU	yes

Table S2. Information for populations phenotyped for floral dimension traits in the field. CAW: Carolyn Wessinger; KANU: R.L. McGregor Herbarium at University of Kansas.

**Population**	**Species**	**Locality**	**Collector**	**Herbarium deposited**
CAW015	*P. virgatus*	Victor, CO	CAW	KANU
CAW017	*P. virgatus*	Lake George, CO	CAW	KANU
CAW019	*P. virgatus*	Buena Vista, CO	CAW	KANU
CAW020	*P. virgatus*	Leadville, CO	CAW	KANU
CAW030	*P. barbatus*	Gila NF, NM	CAW	KANU
CAW031	*P. virgatus*	Cloudcroft, NM	CAW	KANU
CAW032	*P. virgatus*	Ski Cloudcroft, NM	CAW	KANU
CAW033	*P. virgatus*	Alto, NM	CAW	KANU
CAW035	*P. virgatus*	Corona, NM	CAW	KANU
CAW036	*P. barbatus*	Valle Caldera, NM	CAW	KANU
CAW037	*P. barbatus*	Apache Canyon, NM	CAW	KANU
CAW041	*P. barbatus*	Questa, NM	CAW	KANU
CAW080	*P. laevis*	Mt. Carmel Jct, UT	CAW	KANU
CAW162	*P. barbatus*	Lake Mary, AZ	CAW	KANU
CAW164	*P. barbatus*	Mogollon Rim, AZ	CAW	KANU
CAW167	*P. virgatus*	Overgaard, AZ	CAW	KANU
CAW169	*P. barbatus*	Greer, AZ	CAW	KANU
CAW174	*P. barbatus*	Mogollon, NM	CAW	KANU
CAW176	*P. barbatus*	Pinos Altos, NM	CAW	KANU
CAW177	*P. barbatus*	High Rolls, NM	CAW	KANU
CAW180	*P. laevis*	Rosy Canyon, AZ	CAW	KANU
CAW181	*P. eatonii*	Gooseberry Mesa, UT	CAW	KANU
CAW232	*P. eatonii*	Kolob Canyon, UT	CAW	KANU
CAW233	*P. laevis*	Red Hollow Canyon, UT	CAW	KANU
CAW234	*P. eatonii*	Little Creek Mesa, UT	CAW	KANU

Table S3. Results of phylogenetic ANOVA models implemented in *caper* testing the effect of pollination syndrome on floral trait variation. Lambda refers to the estimated Pagel's lambda parameter.

**Model**	**lambda**	**DF**	**F-value**	**p-value**
long stamen length ~ syndrome	0	28	53.949	5.353 e -08
short stamen length ~ syndrome	0.54	28	65.931	7.696 e -09
style length ~ syndrome	0.48	28	44.726	2.937 e -07
tube length ~ syndrome	0	28	59.01	2.282 e -08
tube width ~ syndrome	0.70	28	49.212	1.25 e -07
log nectar volume ~ syndrome	0	28	31.35	5.411 e -06
log nectary area ~ syndrome	1	28	58.258	2.582 e -08
length/width ratio ~ syndrome	0.16	28	302.04	< 2.2 e -16
anthocyanin ~ syndrome	1	28	619.57	< 2.2 e -16
PC1 ~ syndrome	0.46	28	141.76	1.792 e -12
PC2 ~ syndrome	0.44	28	3.0033	0.0941
adjusted hue ~ syndrome	0	23	309.61	7.73 e -15
chroma ~ syndrome	0	23	153.68	9.475 e -12
brightness ~ syndrome	0	23	15.032	0.000763
chromatic contrast bee ~ syndrome	0	23	57.507	1.061 e -07
chromatic contrast bird ~ syndrome	0	23	112.83	2.42 e -10

Table S4. Results of phylogenetic ANOVA models implemented in *caper* testing whether floral traits in bee syndrome species differ according to whether or not the accession is a sister taxon to hummingbird syndrome species. Lambda refers to the estimated Pagel's lambda parameter.

**Model**	**DF**	**lambda**	**F-value**	**p-value**
long stamen length ~ sister status	19	1	2.2707	0.1483
short stamen length ~ sister status	19	1	3.713	0.0691
style length ~ sister status	19	1	2.4001	0.1378
tube length ~ sister status	19	0	0.4426	0.5139
tube width ~ sister status	19	1	0.8223	0.3759
log nectar volume ~ sister status	19	0	1.9712	0.1765
log nectary area ~ sister status	19	0	0.4395	0.5153
length / width ratio ~ sister status	19	0	1.7317	0.2038
anthocyanin ~ sister status	19	1	2.521	0.1288
PC1 ~ sister status	19	0.6	2.7666	0.1127
PC2 ~ sister status	19	0	2.4233	0.136

Table S5. Phylogenetic correlations among traits in bee syndrome species estimated in *caper*.

**Model**	**Correlation coefficient**	**p-value**
Long stamen length, short stamen length	0.9684	6.403 e -13
Long stamen length, style length	0.8671	3.634 e -07
Long stamen length, tube length	0.8608	5.051 e -07
Long stamen length, tube width	0.2111	0.3584
Long stamen length, log nectar volume	0.5232	0.0149
Long stamen length, long nectary area	0.4570	0.0373
Short stamen length, style length	0.8481	1.201 e -06
Short stamen length, tube length	0.8621	5.065 e -07
Short stamen length, tube width	0.2983	0.189
Short stamen length, log nectar volume	0.5621	0.0080
Short stamen length, log nectary area	0.4211	0.0573
Style length, tube length	0.8141	7.094 e -06
Style length, tube width	0.2427	0.2892
Style length, log nectar volume	0.6149	0.0030
Style length, log nectary area	0.3457	0.1248
Tube length, tube width	0.3140	0.1573
Tube length, log nectar volume	0.5705	0.0069
Tube length, log nectary area	0.3894	0.0810
Tube width, log nectar volume	0.4189	0.0587
Tube width, log nectary area	0.3564	0.1128
Log nectar volume, log nectary area	0.5406	0.0114

Table S6. Statistical comparisons of RND distributions between taxa in a 5-taxon testing framework. Reference genome used for analyses was *P. eatonii*.

**B1 taxon**	**H1 taxon**	**H2 taxon**	**B2 taxon**	**Comparison**	**Wilcoxon rank sum statistic**	**p-value**
P. glaber	*P. cardinalis*	*P. eatonii*	*P. laevis*	H1H2 vs. H1B2	8973	0.3772
P. glaber	*P. cardinalis*	*P. eatonii*	*P. laevis*	H1H2 vs. H2B1	14994	0.1215
P. glaber	*P. cardinalis*	*P. eatonii*	*P. laevis*	H1H2 vs. H1B1	299681	4.273 e -11
P. glaber	*P. cardinalis*	*P. eatonii*	*P. laevis*	H1H2 vs. H2B2	685778	< 2.2 e -16
P. glaber	*P. cardinalis*	*P. barbatus*	*P. virgatus*	H1H2 vs. H1B2	48222	0.0005119
P. glaber	*P. cardinalis*	*P. barbatus*	*P. virgatus*	H1H2 vs. H2B1	93241	0.7091
P. glaber	*P. cardinalis*	*P. barbatus*	*P. virgatus*	H1H2 vs. H1B1	699720	2.23 e -8
P. glaber	*P. cardinalis*	*P. barbatus*	*P. virgatus*	H1H2 vs. H2B2	1477880	< 2.2 e -16
P. glaber	*P. cardinalis*	*P. labrosus*	*P.putus/deaveri*	H1H2 vs. H1B2	15374	0.9145
P. glaber	*P. cardinalis*	*P. labrosus*	*P.putus/deaveri*	H1H2 vs. H2B1	18553	0.4028
P. glaber	*P. cardinalis*	*P. labrosus*	*P.putus/deaveri*	H1H2 vs. H1B1	415638	6.818 e -12
P. glaber	*P. cardinalis*	*P. labrosus*	*P.putus/deaveri*	H1H2 vs. H2B2	908752	< 2.2 e -16
P. laevis	*P. eatonii*	*P. barbatus*	*P. virgatus*	H1H2 vs. H1B2	24168	0.361
P. laevis	*P. eatonii*	*P. barbatus*	*P. virgatus*	H1H2 vs. H2B1	18315	0.09904
P. laevis	*P. eatonii*	*P. barbatus*	*P. virgatus*	H1H2 vs. H1B1	916161	< 2.2 e -16
P. laevis	*P. eatonii*	*P. barbatus*	*P. virgatus*	H1H2 vs. H2B2	646253	< 2.2 e -16
P. laevis	*P. eatonii*	*P. labrosus*	*P.putus/deaveri*	H1H2 vs. H1B2	24843	0.01205
P. laevis	*P. eatonii*	*P. labrosus*	*P.putus/deaveri*	H1H2 vs. H2B1	28031	0.9198
P. laevis	*P. eatonii*	*P. labrosus*	*P.putus/deaveri*	H1H2 vs. H1B1	985176	< 2.2 e -16
P. laevis	*P. eatonii*	*P. labrosus*	*P.putus/deaveri*	H1H2 vs. H2B2	812050	< 2.2 e -16
P.virgatus	*P. barbatus*	*P. labrosus*	*P.putus/deaveri*	H1H2 vs. H1B2	92016	0.0002475
P.virgatus	*P. barbatus*	*P. labrosus*	*P.putus/deaveri*	H1H2 vs. H2B1	139307	0.002765
P.virgatus	*P. barbatus*	*P. labrosus*	*P.putus/deaveri*	H1H2 vs. H1B1	1014506	< 2.2 e -16
P.virgatus	*P. barbatus*	*P. labrosus*	*P.putus/deaveri*	H1H2 vs. H2B2	1291339	< 2.2 e -16

Table S7. Phylogenetic correlations among traits in bee syndrome species estimated in *caper*, using the *P. barbatus* reference genome.

**Model**	**Correlation coefficient**	**p-value**
Long stamen length, short stamen length	0.9681	7.002 e -13
Long stamen length, style length	0.8690	3.212 e -07
Long stamen length, tube length	0.8600	5.816 e -07
Long stamen length, tube width	0.2061	0.3701
Long stamen length, log nectar volume	0.524	0.0148
Long stamen length, long nectary area	0.4602	0.0358
Short stamen length, style length	0.8505	1.0429 e -06
Short stamen length, tube length	0.8616	5.225 e -07
Short stamen length, tube width	0.2953	0.1937
Short stamen length, log nectar volume	0.5642	0.0077
Short stamen length, log nectary area	0.4239	0.0558
Style length, tube length	0.8147	6.921 e -06
Style length, tube width	0.2372	0.3006
Style length, log nectar volume	0.6115	0.0032
Style length, log nectary area	0.3502	0.1197
Tube length, tube width	0.3167	0.1619
Tube length, log nectar volume	0.5713	0.0068
Tube length, log nectary area	0.3915	0.0793
Tube width, log nectar volume	0.4180	0.0593
Tube width, log nectary area	0.3575	0.1116
Log nectar volume, log nectary area	0.5435	0.0109

Table S8. Results of phylogenetic ANOVA models implemented in *caper* testing the effect of pollination syndrome on floral trait variation, using the *P. barbatus* reference genome. Lambda refers to the estimated Pagel's lambda parameter.

**Model**	**lambda**	**DF**	**F-value**	**p-value**
long stamen length ~ syndrome	0	28	53.949	5.353 e -08
short stamen length ~ syndrome	0.55	28	65.668	8.009 e -09
style length ~ syndrome	0.49	28	44.413	3.124 e -07
tube length ~ syndrome	0	28	59.01	2.282 e -08
tube width ~ syndrome	0.69	28	48.652	1.387 e -07
log nectar volume ~ syndrome	0	28	31.35	5.411 e -06
log nectary area ~ syndrome	1	28	57.963	2.711 e -08
length / width ratio ~ syndrome	0.15	28	301.73	< 2.2 e -16
anthocyanin ~ syndrome	1	28	617.75	< 2.2 e -16
PC1 ~ syndrome	0.44	28	141.68	1.804 e -12
PC2 ~ syndrome	0.43	28	3.0295	0.09275
adjusted hue ~ syndrome	0.94	23	302.18	1.003 e -14
chroma ~ syndrome	0	23	156.68	9.475 e -12
brightness ~ syndrome	0	23	15.032	0.000763
chromatic contrast bee ~ syndrome	0	23	57.507	1.061 e -07
chromatic contrast bird ~ syndrome	0	23	112.83	2.42 e -10

Table S9. Results of phylogenetic ANOVA models implemented in *caper* testing whether floral traits in bee syndrome species differ according to whether or not the accession is a sister taxon to hummingbird syndrome species, using the *P. barbatus* reference genome. Lambda refers to the estimated Pagel's lambda parameter.

**Model**	**DF**	**lambda**	**F-value**	**p-value**
long stamen length ~ sister status	19	1	2.3794	0.1394
short stamen length ~ sister status	19	1	3.8259	0.0653
style length ~ sister status	19	1	2.5132	0.1294
tube length ~ sister status	19	0	0.4426	0.5139
tube width ~ sister status	19	1	0.9375	0.3451
log nectar volume ~ sister status	19	0	1.9712	0.1765
log nectary area ~ sister status	19	0	0.4395	0.5153
length / width ratio ~ sister status	19	0	1.7317	0.2038
anthocyanin ~ sister status	19	1	2.5258	0.1285
PC1 ~ sister status	19	0.7	2.6172	0.1222
PC2 ~ sister status	19	0	2.4233	0.136

Table S10. Statistical comparisons of RND distributions between taxa in a 5-taxon testing framework. Reference genome used for analyses was *P. barbatus*.

**B1 taxon**	**H1 taxon**	**H2 taxon**	**B2 taxon**	**Comparison**	**Wilcoxon rank sum statistic**	**p-value**
P. glaber	*P. cardinalis*	*P. eatonii*	*P. laevis*	H1H2 vs. H1B2	12522	0.7515
P. glaber	*P. cardinalis*	*P. eatonii*	*P. laevis*	H1H2 vs. H2B1	23602	0.3671
P. glaber	*P. cardinalis*	*P. eatonii*	*P. laevis*	H1H2 vs. H1B1	451140	6.43 e -15
P. glaber	*P. cardinalis*	*P. eatonii*	*P. laevis*	H1H2 vs. H2B2	1024847	< 2.2 e -16
P. glaber	*P. cardinalis*	*P. barbatus*	*P. virgatus*	H1H2 vs. H1B2	72199	0.0006686
P. glaber	*P. cardinalis*	*P. barbatus*	*P. virgatus*	H1H2 vs. H2B1	139390	.2658
P. glaber	*P. cardinalis*	*P. barbatus*	*P. virgatus*	H1H2 vs. H1B1	1197275	4.56 e -14
P. glaber	*P. cardinalis*	*P. barbatus*	*P. virgatus*	H1H2 vs. H2B2	2699681	< 2.2 e -16
P. glaber	*P. cardinalis*	*P. labrosus*	*P.putus/deaveri*	H1H2 vs. H1B2	21447	0.8419
P. glaber	*P. cardinalis*	*P. labrosus*	*P.putus/deaveri*	H1H2 vs. H2B1	25371	0.6417
P. glaber	*P. cardinalis*	*P. labrosus*	*P.putus/deaveri*	H1H2 vs. H1B1	415638	6.818 e -12
P. glaber	*P. cardinalis*	*P. labrosus*	*P.putus/deaveri*	H1H2 vs. H2B2	908752	< 2.2 e -16
P. laevis	*P. eatonii*	*P. barbatus*	*P. virgatus*	H1H2 vs. H1B2	41996	0.08567
P. laevis	*P. eatonii*	*P. barbatus*	*P. virgatus*	H1H2 vs. H2B1	27932	0.08469
P. laevis	*P. eatonii*	*P. barbatus*	*P. virgatus*	H1H2 vs. H1B1	1471736	< 2.2 e -16
P. laevis	*P. eatonii*	*P. barbatus*	*P. virgatus*	H1H2 vs. H2B2	1126924	< 2.2 e -16
P. laevis	*P. eatonii*	*P. labrosus*	*P.putus/deaveri*	H1H2 vs. H1B2	40892	0.1303
P. laevis	*P. eatonii*	*P. labrosus*	*P.putus/deaveri*	H1H2 vs. H2B1	53938	0.1573
P. laevis	*P. eatonii*	*P. labrosus*	*P.putus/deaveri*	H1H2 vs. H1B1	1694462	< 2.2 e -16
P. laevis	*P. eatonii*	*P. labrosus*	*P.putus/deaveri*	H1H2 vs. H2B2	1455412	< 2.2 e -16
P.virgatus	*P. barbatus*	*P. labrosus*	*P.putus/deaveri*	H1H2 vs. H1B2	163296	6.843 e -07
P.virgatus	*P. barbatus*	*P. labrosus*	*P.putus/deaveri*	H1H2 vs. H2B1	232637	5.015 e -06
P.virgatus	*P. barbatus*	*P. labrosus*	*P.putus/deaveri*	H1H2 vs. H1B1	1707757	< 2.2 e -16
P.virgatus	*P. barbatus*	*P. labrosus*	*P.putus/deaveri*	H1H2 vs. H2B2	2121034	< 2.2 e -16

Supplemental References

Andrews, S., Krueger, F., Segonds-Pichon, A., Biggins, L., Krueger, C., & Wingett, S. (2010). FastQC. *A quality control tool for high throughput sequence data*, *370*.

Chen, S., Zhou, Y., Chen, Y., & Gu, J. (2018). fastp: an ultra-fast all-in-one FASTQ preprocessor. *Bioinformatics*, *34*(17), i884-i890.

Danecek, P., Auton, A., Abecasis, G., Albers, C. A., Banks, E., DePristo, M. A., Handsaker, R. E., Lunter, G., Marth, G. T., & Sherry, S. T. (2011). The variant call format and VCFtools. *Bioinformatics*, *27*(15), 2156-2158.

Harborne, J. (1984). *Phytochemical methods 2nd ed*. Chapman and Hall.

Jarvis, D. E., Stevens, M. R., Carter, P., Lin, Y. F., Jaggi, K. E., Jijon, G., Kalt, T., Calixto, J., Standring, S., & Torres, K. (2025). Whole-genome assembly and annotation of the firecracker penstemon (Penstemon eatonii). *Journal of Heredity*, *116*(3), 373-381.

Jun, G., Wing, M. K., Abecasis, G. R., & Kang, H. M. (2015). An efficient and scalable analysis framework for variant extraction and refinement from population-scale DNA sequence data. *Genome research*, *25*(6), 918-925.

Li, H. (2011). A statistical framework for SNP calling, mutation discovery, association mapping and population genetical parameter estimation from sequencing data. *Bioinformatics*, *27*(21), 2987-2993.

Li, H. (2013). Aligning sequence reads, clone sequences and assembly contigs with BWA-MEM. *arXiv preprint arXiv:1303.3997*.

Li, H., Handsaker, B., Wysoker, A., Fennell, T., Ruan, J., Homer, N., Marth, G., Abecasis, G., & Durbin, R. (2009). The sequence alignment/map format and SAMtools. *Bioinformatics*, *25*(16), 2078-2079.

Maia, R., Gruson, H., Endler, J. A., & White, T. E. (2019). pavo 2: new tools for the spectral and spatial analysis of colour in R. *Methods in Ecology and Evolution*, *10*(7), 1097-1107.

Minh, B. Q., Hahn, M. W., & Lanfear, R. (2020). New methods to calculate concordance factors for phylogenomic datasets. *Molecular biology and evolution*, *37*(9), 2727-2733.

Nguyen, L.-T., Schmidt, H. A., Von Haeseler, A., & Minh, B. Q. (2015). IQ-TREE: a fast and effective stochastic algorithm for estimating maximum-likelihood phylogenies. *Molecular biology and evolution*, *32*(1), 268-274.

Schindelin, J., Arganda-Carreras, I., Frise, E., Kaynig, V., Longair, M., Pietzsch, T., Preibisch, S., Rueden, C., Saalfeld, S., & Schmid, B. (2012). Fiji: an open-source platform for biological-image analysis. *Nature methods*, *9*(7), 676-682.

Smith, S. A., & O'Meara, B. C. (2012). treePL: divergence time estimation using penalized likelihood for large phylogenies. *Bioinformatics*, *28*(20), 2689-2690.

Smith, S. D. (2014). Quantifying color variation: improved formulas for calculating hue with segment classification. *Applications in Plant Sciences*, *2*(3), 1300088.

Stevens, J. T., Wheeler, L. C., Williams, N. H., Norton, A. M., & Wessinger, C. A. (2023). Predictive links between petal color and pigment quantities in natural Penstemon hybrids. *Integrative and Comparative Biology*, *63*(6), 1340-1351.

Stone, B. W., & Wessinger, C. A. (2024). Ecological diversification in an adaptive radiation of plants: the role of de novo mutation and introgression. *Molecular biology and evolution*, *41*(1), msae007.

Vorobyev, M., & Osorio, D. (1998). Receptor noise as a determinant of colour thresholds. *Proceedings of the Royal Society of London. Series B: Biological Sciences*, *265*(1394), 351-358.

Wessinger, C. A., & Rausher, M. D. (2015). Ecological transition predictably associated with gene degeneration. *Molecular biology and evolution*, *32*(2), 347-354.

Zhang, C., Rabiee, M., Sayyari, E., & Mirarab, S. (2018). ASTRAL-III: polynomial time species tree reconstruction from partially resolved gene trees. *BMC bioinformatics*, *19*(6), 153.
